# SETD3 is a positive regulator of DNA-damage-induced apoptosis

**DOI:** 10.1038/s41419-019-1328-4

**Published:** 2019-01-25

**Authors:** Elina Abaev-Schneiderman, Lee Admoni-Elisha, Dan Levy

**Affiliations:** 1 The Shraga Segal Department of Microbiology, Immunology and Genetics, Be’er-Sheva, 84105 Israel; 2 National Institute for Biotechnology in the Negev, Ben-Gurion University of the Negev, P.O.B. 653, Be’er-Sheva, 84105 Israel

## Abstract

SETD3 is a member of the protein lysine methyltransferase (PKMT) family, which catalyzes the addition of methyl group to lysine residues. However, the protein network and the signaling pathways in which SETD3 is involved remain largely unexplored. In the current study, we show that SETD3 is a positive regulator of DNA-damage-induced apoptosis in colon cancer cells. Our data indicate that depletion of SETD3 from HCT-116 cells results in a significant inhibition of apoptosis after doxorubicin treatment. Our results imply that the positive regulation is sustained by methylation, though the substrate remains unknown. We present a functional cross-talk between SETD3 and the tumor suppressor p53. SETD3 binds p53 in cells in response to doxorubicin treatment and positively regulates p53 target genes activation under these conditions. Mechanistically, we provide evidence that the presence of SETD3 and its catalytic activity is required for the recruitment of p53 to its target genes. Finally, Kaplan–Meier survival analysis, of two-independent cohorts of colon cancer patients, revealed that low expression of SETD3 is a reliable predictor of poor survival in these patients, which correlates with our findings. Together, our data uncover a new role of the PKMT SETD3 in the regulation of p53-dependent activation of apoptosis in response to DNA damage.

## Introduction

Apoptosis is a conserved and essential cellular process of programmed cell death which allows damaged cells removal, thus maintaining and regulating homeostasis in multicellular organisms^1^. DNA-damage-induced agents such as chemotherapeutic drugs and irradiation can lead to apoptotic death through a *p53*-dependent pathway^2^. The transcription factor p53 is an established tumor suppressor, which is activated upon DNA damage. Following activation, p53 induces the transcription of many pro-apoptotic genes, such as BAX^3^, PUMA^4^, and NoxA^5^. The activation of these target genes results in a cascade of downstream events, which involves mitochondrial outer membrane permeabilization, cytochrome c release followed by the activation of caspases that eventually causes cell death^6^. Any aberration of the apoptotic pathway is potentially tumorigenic and may lead to treatment resistance^2,7^.

Lysine methylation is catalyzed by members of the protein lysine methyltransferases (PKMTs) family. There are over 60 candidate members of this enzyme family, the vast majority of which contain a conserved SET domain that is responsible for their enzymatic activity^8^. Little is known about the PKMT SETD3, though it is abundantly expressed in many tissues, including muscle, where it promotes myocyte-differentiation by regulating the transcription of muscle-related genes^9^. We have recently shown that SETD3 methylates the transcription factor FoxM1 to regulate VEGF expression under normoxic and hypoxic conditions^10^. Similar results were also observed during hypoxic pulmonary hypertension in rats^11^. In addition, SETD3 was shown to interact with PCNA, Maf1, and RNF7, which are involved in DNA replication^12^, hence, linking SETD3 to DNA replication and DNA repair.

Recent papers have linked the expression of SETD3 to oncogenic processes^13,14^. For instance, high expression of SETD3, which lacks the SET domain, displays oncogenic properties in lymphoma^14^, similarly, in liver cancer, the upregulation of SETD3 is associated with cancer development^15^. Thus, suggesting a pro-tumorigenic role of SETD3. However, on the contrary, SETD3 was also suggested as a biomarker for renal cancer diagnosis^16^. Where low expression levels of SETD3 were associated with significantly shorter disease-free survival^16^. In zebrafish models, overexpression of SETD3 decreased cell viability and induced apoptosis^17^. Consequently, implying an opposite, anti-tumorigenic role. It seems therefore, that SETD3’s roles and involvement in cancer may vary depending on its type.

Bioinformatics database analysis revealed that high expression of SETD3 correlates with higher survival rate in colon cancer patients. To further explore the cellular roles of SETD3 in colon cancer, we immunoprecipitated SETD3 from HCT-116 cells followed by mass spectrometry and identified 215 novel interacting proteins showing a significant link to DNA damage response pathways. Depletion of SETD3 in cells results in inhibition of apoptosis in response to doxorubicin (DOX), Etoposide or Abiplatin treatment. Our data suggest a functional cross talk between SETD3 and p53. SETD3 doesn’t bind nor methylates p53 in vitro. However, we show that p53 and SETD3 physically interact in cells and that SETD3 positively regulates the activation of p53 target genes following DOX treatment. ChIP experiments revealed that SETD3 catalytic activity is required for the recruitment of p53 to its target genes. Together, our data uncover new roles of the PKMT SETD3 in the regulation of p53-dependent activation of apoptosis in response to DNA damage.

## Results

### SETD3 is a potential regulator of DNA damage

**Fig. 1:**
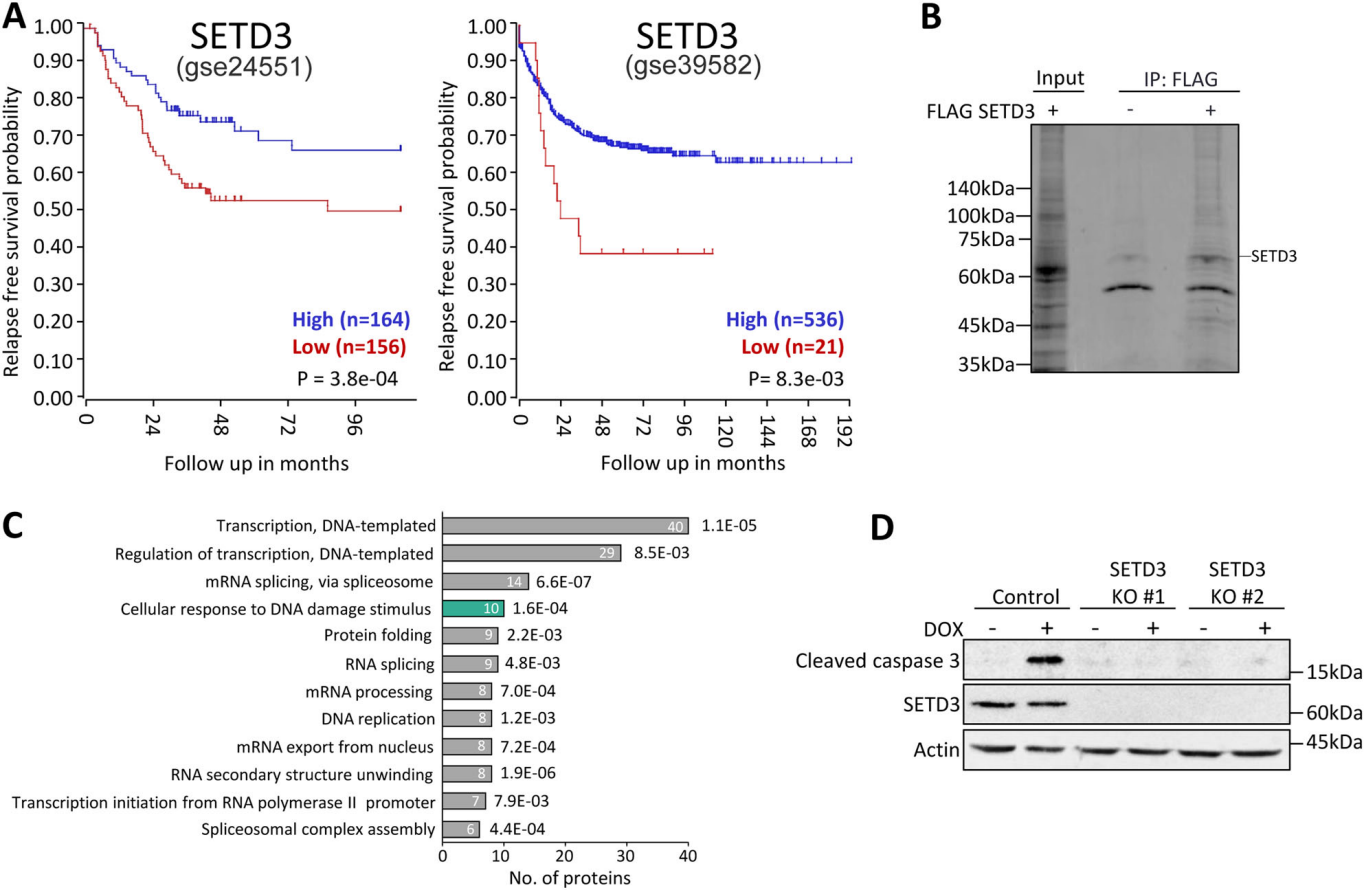
SETD3 interacts with DNA-damage associated proteins. **a** Kaplan–Meier survival recurrence-free survival of 333 (gse24551) (Left) and of 566 (gse39582) (Right) colorectal cancer patients. High SETD3 expression represented in the blue line, while low expression of SETD3 is in red. **b** Coomassie staining of HCT-116 cells lysate ± overexpression of FLAG-SETD3 followed by immunoprecipitation with FLAG antibody conjugated beads. Input lane is of FLAG-SETD3 overexpressed cell lysate. **c** GO biological processes analysis of proteins identified in the mass spectrometry experiment. **d** HCT-116 control and SETD3 KO cells treated with DOX, followed by western blot with the indicated antibodies

We utilized the R2 Genomics Analysis and Visualization Platform clinical data (http://r2.amc.nl) derived from whole-genome expression of the survival rate of colorectal cancer patients with high and low expression of SETD3 (Fig. 1a). Kaplan–Meier survival analysis from two-independent cohorts revealed that high expression of SETD3 may be a potential predictor of overall survival in these patients. These results suggest that SETD3 has positive clinical significance in colorectal cancer. To study the cellular role of SETD3 in colorectal carcinoma cells (HCT-116), we immunoprecipitated overexpressed FLAG-SETD3 from the nuclear fraction followed by mass spectrometry (Fig. 1b). In all, 215 proteins were identified as SETD3 interacting proteins. Gene ontology (GO) analysis of these proteins showed enrichment of many biological processes. One of the most significant processes was DNA-damage response (Fig. 1c). The full list of the newly identified SETD3 interacting proteins is shown in Supplementary File 1.

### SETD3-positively regulates DNA-damage-induced apoptosis

DNA-damage response is tightly linked to the induction of apoptosis^18^. We therefore postulated that SETD3 might be involved in the regulation of this process. One of the most important anti-tumorigenic agents for solid tumors Doxorubicin (DOX)^19^ is an intercalating agent which inhibits topoisomerase and induces apoptosis via the activation of caspases and disruption of mitochondrial membrane potential^20^. Consequently, we examined the expression of cleaved caspase 3 following treatment with DOX in control and in two SETD3 knockout HCT-116 cells generated by the CRISPR-cas9 system derived from single clones of two-independent gRNAs (Fig. 1d). The results clearly demonstrate that cleaved caspase 3 expressions was induced following DOX treatment in the control cells and was completely lost in the SETD3 KO cells, suggesting that SETD3 is a positive regulator of apoptosis in response to DOX treatment.

**Fig. 2:**
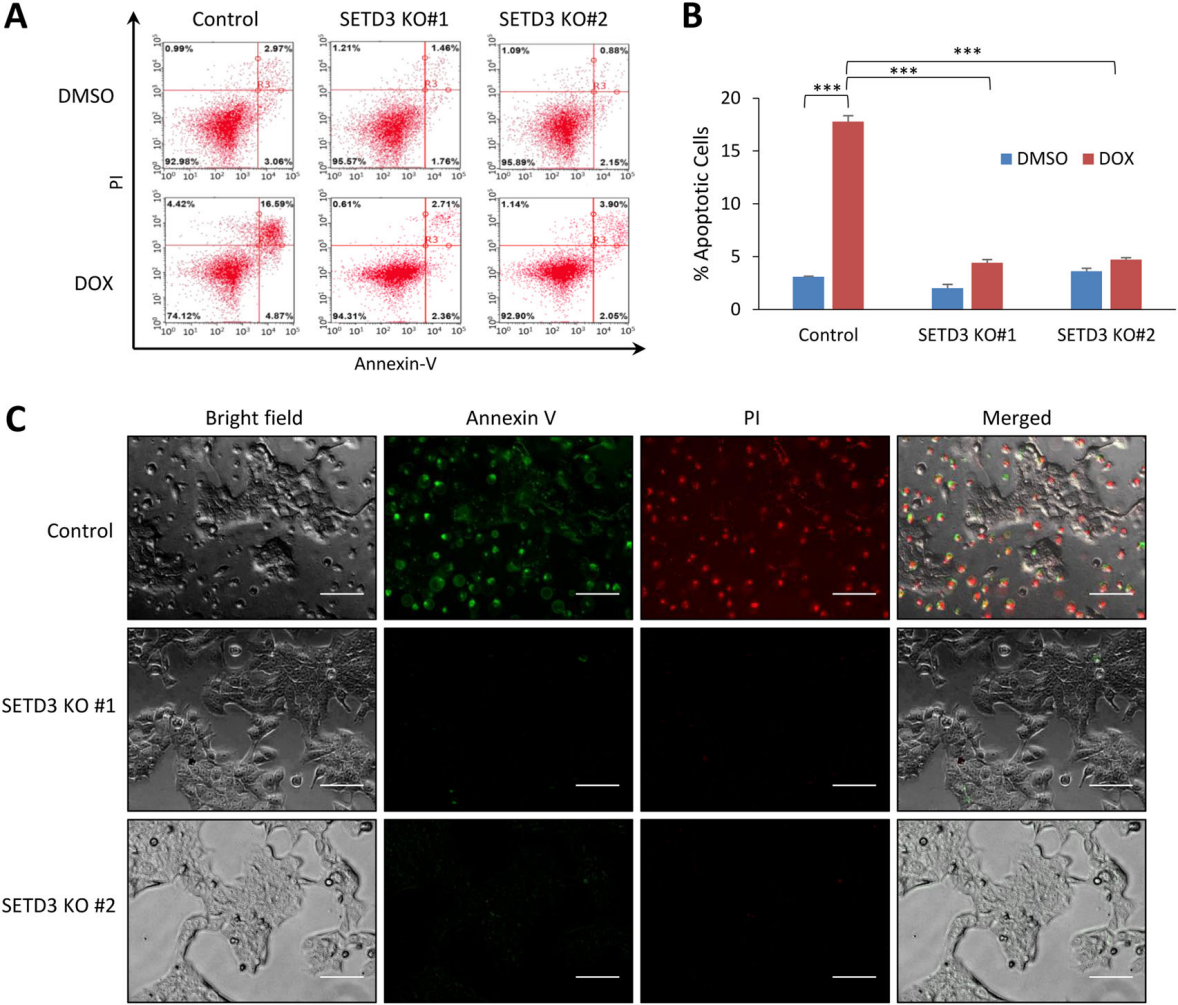
SETD3 positively regulates DOX-induced apoptosis. **a** FACS analysis of KO and control cells post DOX or vehicle treatment. **b** Quantification of apoptotic cells percentage of three-independent FACS analyses, ***p ≤ 0.001. **c** Control and KO cells were stained with FITC-Annexin V and PI post treatment with DOX and viewed under fluorescent microscope (scale bar signifies 100 µM, pictures were taken under ×20 magnification)

We next sought to determine the apoptotic phenotype following DOX treatment in control and SETD3-KO HCT-116 cells by FACS analysis using FITC-Annexin-V and propidium iodide (PI) staining (Fig. 2a and quantitation in Fig. 2b). Following treatment, control cells underwent apoptosis. However, the SETD3 KO cells were barely damaged and only slight elevation in Annexin-V and PI staining was observed (Fig. 2a). Similar results were obtained in response to Etoposide (Figure S3A) and Abiplatin treatments (Figure S3C). Quantification of these experiments is presented in figures S3B and S3D, respectively, indicating that this phenotype is not restricted to DOX. Consistent results were obtained under the microscope. As seen in Fig. 2c, Annexin-V and PI staining appeared only in control cells and no staining was detected in both SETD3 KO cells after DOX treatment. Images of the untreated cells are shown in Figure S1. These results imply that SETD3 has an imperative role in the ability of the cells to undergo apoptosis after DNA-damage treatment.

### Doxorubicin-induced apoptosis is SETD3 and methylation dependent

**Fig. 3:**
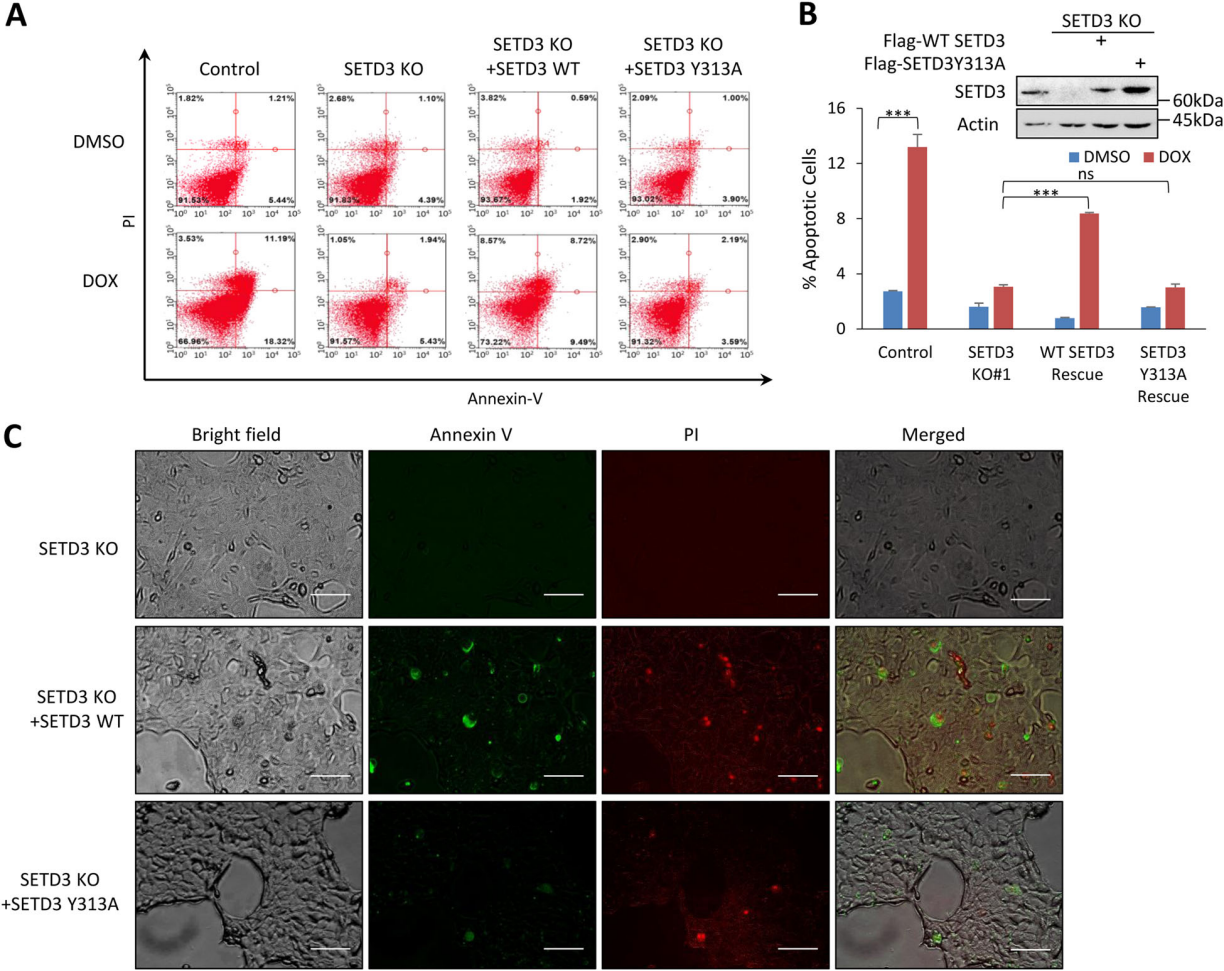
DNA-damage-induced apoptosis is SETD3 and methylation dependent. **a** FACS analysis of control, KO, WT SETD3 rescue and catalytic inactive (Y313A) SETD3 rescue cells, post DOX or vehicle treatment. **b** Quantification of apoptotic cells percentage of three-independent FACS analyses, ***p ≤ 0.001, ns *p* > 0.05. Western blot of the control, KO and rescued cells lysate. **c** SETD3 KO and rescued (with WT or Y313A) cells were stained with FITC-Annexin V and PI post treatment with DOX and viewed under fluorescent microscope (scale bar signifies 100 µM, pictures were taken under ×20 magnification)

In order to test whether the observed phenotype is SETD3 dependent, we measured apoptosis rate post DOX treatment in SETD3 KO cells rescued by Flag-SETD3 wild type (WT). As shown in Fig. 3a (quantitation in Fig. 3b), similar to control cells, the rescued cells can partially regain their ability to undergo DNA-damage-induced apoptosis. Next, we sought to determine whether methylation is required to obtain this phenotype. To this end, we rescued the KO cells with catalytic inactive SETD3 by FLAG-SETD3 Y313A. As shown in Fig. 3a, b, SETD3 Y313A cells did not undergo apoptosis as did the control and the SETD3 WT rescued cells. In a similar setup, SETD3 WT and SETD3 Y313A were overexpressed in the SETD3 KO cells, treated with DOX and the intensity of Annexin V and PI was visualized by fluorescent microscopy (Fig. 3c). Consistent with our previous findings, apoptosis was not induced in the SETD3 KO cells and was partially rescued by WT SETD3 but not by the SETD3 Y313A (Fig. 3c and Figure S2, using SETD3 KO#2 cell line). Taken together, the data suggest that the induction of apoptosis following DNA-damage treatment is not only SETD3 dependent, but also methylation dependent.

### SETD3 positively regulates the activation of p53-induced apoptosis target genes

**Fig. 4:**
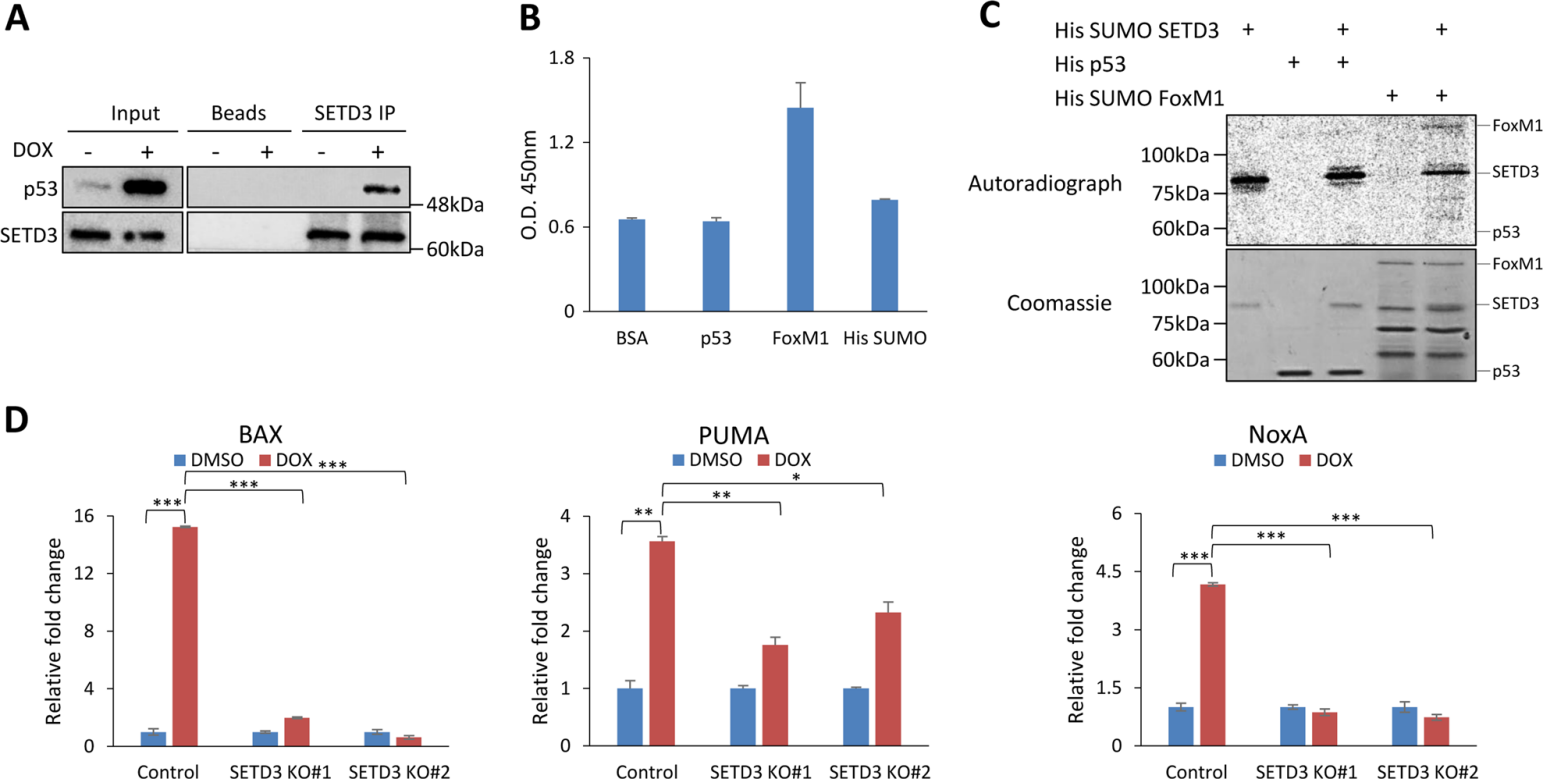
SETD3-positively regulates the activation of DNA-damage-induced apoptosis target genes. **a** Immunoprecipitation of endogenous SETD3 from whole-cell lysate (HCT-116 cells) followed by western blot with the indicated antibodies. **b** ELISA experiment between His-p53 and His-SUMO SETD3, His-SUMO FoxM1 used as positive control. **c** In vitro methylation assay between His-SUMO SETD3 and His-p53. Using His-SUMO FoxM1 as positive control. **d** qPCR analysis of extracted mRNA from HCT-116 control and KO cells, as indicated, post DOX or vehicle treatment. Graphs show the relative expression levels (normalized to GAPDH) of the indicated genes. *p ≤ 0.05, **p ≤ 0.01, ***p ≤ 0.001

We next wanted to understand the underlying mechanism of SETD3 role in the induction of apoptosis following DOX treatment. Given the pivotal role of p53 in the induction of apoptosis in response to DNA damage, we hypothesized that there is a potential functional cross-talk between p53 and SETD3. In a co-immunoprecipitation experiment we found that endogenous SETD3 binds p53 after DOX treatment (Fig. 4a). To test whether this is a direct interaction, we performed an ELISA experiment using recombinant His-SUMO SETD3 and His-p53. No direct interaction between the two was found (Fig. 4b). In addition, In vitro methylation assay using recombinant purified proteins revealed that SETD3 does not methylate p53 (Fig. 4c). Recombinant His-SUMO FoxM1 served as a positive control for the in vitro reactions^10^. Taken together, our data may suggest that SETD3 participates in p53 apoptosis pathway following DNA damage, but does not bind nor methylates p53 in vitro.

To examine a potential role of SETD3 in regulating the expression of p53 target genes we measured the mRNA expression of BAX^3^, PUMA^4^, and NoxA^5^ in control and SETD3 KO cells treated with DOX or with vehicle (Fig. 4d). As expected, DOX treatment led to a significant increase of these target genes in the control cells. Strikingly, their expression was significantly downregulated in the SETD3 KO cells. These results suggest that SETD3-positively regulates apoptosis via the regulation of p53 target genes transcription.

### SETD3 is required for p53 recruitment to its target genes

**Fig. 5:**
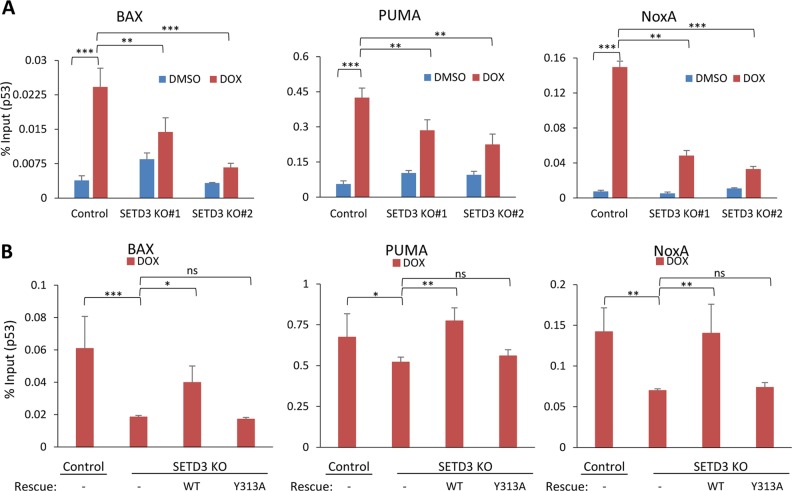
SETD3 catalytic activity is required for p53 recruitment to its target genes following DOX treatment. **a** Chromatin immunoprecipitation (ChIP) assay of control and SETD3 KO HCT-116 cells treated with DOX or vehicle. DNA fragments were immunoprecipitated with p53 antibody. Values were compared to input samples. *p ≤ 0.05, **p ≤ 0.01, ***p ≤ 0.001. **b** Chromatin immunoprecipitation (ChIP) assay of control, SETD3 KO and rescued (with WT or Y313A) cells, post DOX treatment. DNA fragments were immunoprecipitated with p53 antibody. Values were compared to input samples. *p ≤ 0.05, **p ≤ 0.01, ***p ≤ 0.001

Having demonstrated that SETD3-positively regulates p53 transcriptional activity following DNA damage, our working hypothesis was that SETD3 may be required for p53 binding to the promoter area of its target genes. We, therefore, performed a ChIP experiment by precipitating endogenous p53 from control and two KO cells ± DOX treatment (Fig. 5a and Supplementary Figure S4A). A significant enrichment of p53 at the BAX, NoxA and PUMA promoters was observed in the control cells after treatment. Consistent with our hypothesis, significantly lower occupancy of p53 on these target genes was observed in the SETD3 KO cells. To determine whether the catalytic activity of SETD3 is required for p53 recruitment to its target genes, we have rescued the SETD3-KO cells with either SETD3 WT or catalytic inactive, SETD3 Y313A mutant (Fig. 5b and Supplementary Figure S4B). The results demonstrate that while SETD3 WT can partly restore p53 recruitment to its target genes after DOX treatment, the catalytic inactive SETD3 Y313A failed to do so, similarly to the KO cells. We thus conclude, that SETD3 catalytic activity is required to direct p53 to its target genes following DNA damage.

## Discussion

The cellular roles of SETD3 remain largely unexplored. Our data provide evidence that SETD3 is a positive regulator of apoptosis following DNA-damage treatment. In a proteomic screen, to identify new SETD3 interacting proteins, DNA damage was one of the highly significant processes that were enriched. This was surprising, as these experiments were performed under un-stressed conditions. We speculated that our overexpression conditions were sufficient to see DNA-damage-related interactions also under basal conditions.

Our proteomic analysis revealed 215 proteins that specifically interact with SETD3. These are new potential substrates of SETD3, some of which fit the phenotype described in this study and some suggest other roles for SETD3 in cells. Notably, many of these processes are linked to RNA biology, such as splicing, mRNA export and RNA secondary structure (Fig. 1b), emphasizing a potentially significant role of SETD3 in RNA biology. While these wide variety of processes are out of the scope of this particular paper, further research is required to address these important SETD3 cellular roles.

The tumor suppressor p53 is a central player in cellular DNA-damage response and as such it was shown to be regulated by numerous post translational modifications^2,21^, including methylation. p53 methylation at K370, K372, K373, and K382 by the PKMTs SETD7^22^, SMYD2^23^ and SETD8^24^, SETDB1^25^, GLP/G9A^26^ suppresses p53 transcriptional activity in several cancer cell line models, including lung, kidney, and bone. In contrast, SETD7-mediated p53 methylation at K372 has been shown to enhance p53 activity^22^. It was also recently shown that G9A stimulates p53 activity in a methylation-independent mechanism^27^. Our data suggest that SETD3 induces apoptosis via the p53 pathway. The fact that we can rescue the apoptosis phenotype only by SETD3 WT and not by the catalytic inactive SETD3 Y313A suggests that the enzymatic activity of SETD3 is required for DOX-induced apoptosis and p53 recruitment to its target genes. Interestingly, our findings nicely correlate with the work of Chen et al. showing that overexpression of the catalytic inactive (SET domain lacking) SETD3^14^ led to lymphoma progression. The physical interaction between SETD3 and p53 suggested that p53 is a direct target for methylation by SETD3. However, our results indicate that this is not the case. SETD3 doesn’t bind nor methylates p53 in vitro, but is required for p53 recruitment to its target genes. These findings raise a number of additional questions regarding the exact location of SETD3 in the p53-dependent apoptotic pathway and the identification of the substrate that directly interacts and is methylated by SETD3 to induce apoptosis. Future proteomics followed by precise biochemical experiments are required to address this important issue.

Apoptosis is an evolutionarily conserved process which is tightly regulated in various ways^28^. This regulation network is essential in various diseases, however, when it comes to cancer, induction of apoptosis serves also as a therapeutic tool. Many of the existing cancer treatments are based on the cellular ability to undergo apoptosis^29,30^. For example, drugs like DOX^31^, Paclitaxel^32^, and Cisplatin^33^ interfere with DNA repair mechanisms, leading to DNA damage followed by induction of cell cycle arrest or apoptosis. Many oncological patients receive chemotherapy. Unfortunately, some of them develop resistance to these agents and the treatment becomes no longer effective. The cancer cells of these patients harbor numerous aberrations that allow them, for instance, to overcome DNA-damage-induced apoptosis^30^. We found that high expression of SETD3 correlates with better survival of colon cancer patients which highlights the link between our findings to the clinics. Specifically, our work proposes that low levels of SETD3 may serve as a potential diagnostic marker of resistance to DOX treatment in colon cancer patients. In summary, our findings demonstrate that SETD3 is a positive regulator of p53-dependent activation of DOX-induced apoptosis and adds another layer of regulation for p53 activity under these conditions with potential practical therapeutic implications in normal physiological processes and in disease state.

## Material and methods

### Cell lines and transfection

Human colon carcinoma cells (HCT-116) was maintained in Dulbecco’s modified Eagle’s medium (Sigma-Aldrich, D5671) with 10% fetal bovine serum (Gibco), 2 mg/ml l-glutamine (Sigma-Aldrich, G7513), 1% penicillin-streptomycin (Sigma, P0781) and non-essential amino acids (Sigma-Aldrich, M7145), and they were cultured at 37 °C in a humidified incubator with 5% CO_2_. For transient transfection, we used Poly (ethyleneimine) (PEI) solution with the medium as described above or with Mirus LT1 according to the manufacturer protocol.

### DNA-damage treatment

DNA-damage agents used in this study were; DOX (2 µM) (Sigma-Aldrich) dissolved in 100% DMSO (Sigma-Aldrich); Etoposide (30 µM) (TEVA); Abiplatin (60 µM) (TEVA). Cells were subjected to this treatment for 3 h (or with the appropriate vehicle control). After reagent removal, cells were washed once with PBSx1 and released to fresh medium for 24 h and then analyzed.

### Plasmids

Plasmids used for overexpression in cells were: pcDNA-FLAG-SETD3, pcDNA-FLAG-SETD3 Y313A and pcDNA-Empty. For CRISPR/Cas9-mediated gene disruption, two different guide RNAs (gRNAs) of SETD3 exons were sub-cloned to the lentiCRISPR plasmid (Addgene, #49535). gRNA sequences that were used to target SETD3 are: #1- GTATGTGCAGATCCGGACTC, #2- TACAGCAACTGTGTCACCAA. Following transfection and puromycin selection, single clones were isolated, expanded and validated by sequencing. Plasmids used for expression and purification of recombinant proteins were His-Sumo SETD3 and His-sumo FoxM1 previously described in Cohn et al^10^; and His p53 was kindly provided by Eyal Arbely.

### Genomic DNA extraction

For the KO genomic DNA validation, the QuickExtract™ DNA Extraction Solution 1.0 kit was used. Briefly, cells were harvested, extraction solution was added and tubes were vortexed for 15 s. Then, incubated for 6 min at 65 °C, vortexed again and incubated for 2 min at 98 °C. Five microliters was taken for PCR. We used the following primers, KO#1 (gRNA #1) Fw 5′-TTAGGCGCGCCAAGAGTTTTGGGGGGATAGTTT-3′ Rev 5′-GCCTTAATTAAAAAATTGTATCATTTTAGCACCC-3′ and KO#2 (gRNA #2) Fw 5′-TTAGGCGCGCCTTAAGGCAAAGTGGGGAGAGAATA-3′ Rev 5′-GCCTTAATTAAGGAAGCTCACAATTACTTCACCC-3′.

### Recombinant protein expression and purification

*Escherichia coli* BL21 derivative Rosetta host strain, transformed with a plasmid encoding a protein of interest, were grown in LB media. Bacteria were collected by centrifugation after IPTG induction and lysed by sonication on ice (25% amplitude, 1 min total, 10 s on/off). The tagged fusion proteins were purified on His-Trap column using AKTA Pure protein purification system (GE).

### Western blots and antibodies

Primary antibodies used were as follows: SETD3 (ab176582; Abcam), p53 (sc-126; Santa Cruz) Actin (ab3280; Abcam). Secondary HRP-conjugated antibodies (goat anti-mouse and goat anti-rabbit) were from the Jackson ImmunoResearch (115-035-062 and 111-035-144, respectively). Coomassie stain was purchased from Expendon (ISB1L).

### Immunoprecipitation

Cells were lysed in RIPA lysis buffer (50 mM Tris-HCl pH 8, 150 mM NaCl, 1% Nonidet P-40, 0.5% deoxycholate, 0.1% SDS (v/v), 1 mM dithiothreitol (DTT) and Sigma protease inhibitor cocktail (P8340, diluted 1:100)). Lysates were incubated for 1 h at 4 °C with 15 μl protein A/G beads (Santa Cruz Biotechnology) as a pre-clear step. Pre-cleared lysates including were incubated overnight at 4 °C with SETD3 antibody with beads or beads only as a control. After incubation, beads were washed three times with lysis buffer, heated at 95 °C for 5 min in protein sample buffer, and resolved by SDS-PAGE.

### Enzyme-linked immunosorbent assay (ELISA)

ELISA plates (Greiner 96W) were incubated with 2 μg His-p53, His–sumo-FoxM1 (as positive control) and His-SUMO (as negative control) for 1 h at room temperature. The plates were then washed with PBS supplemented with 0.1% Tween® 20 (PBST) and blocked with 3% BSA in PBST for 1 h. Following blocking, the plates were washed and covered with 0.5 μg His-SUMO-SETD3 or BSA protein (negative control) for 1 h. Plates were then washed and incubated with primary antibody (anti-SETD3, 1:10,000 dilution) followed by incubation with secondary HRP-conjugated antibody (goat anti-rabbit, 1:2000 dilution). After adding TMB (3,3′,5,5′-Tetramethylbenzidine) reagent and 1N H_2_SO_4_ (to discontinue the reaction), absorbance at 450 nm was detected using a Tecan Infinite M200 plate reader.

### In vitro methylation assay

Reaction tubes, containing recombinant proteins were incubated overnight at 30 °C with 2mCi H3-labeled S-adenosylmethionine (AdoMet; Perkin-Elmer) in methylation buffer (50 mM Tris-HCl, pH 9, 10% glycerol (v/v), 20 mM KCl and 5 mM MgCl_2_). Reaction mixtures (final volume of 25 µl) were resolved by SDS-PAGE, followed by autoradiography to detect methylation events and Coomassie staining to validate the presence of all proteins in the reaction.

### Samples preparation for mass spectrometry

Endogenous SETD3 was immunoprecipitated from HCT-116 cells after lysis using the MBT Small scale Nuclear Protein Extraction. Briefly, cells were collected and washed with PBSx1, the pellet was suspended in lysis buffer (10 mM HEPES, pH 7.9, 1.5 mM MgCl_2_, 10 mM KCl) including DTT (1:1000) and protein inhibitor (PI) (1:100) and incubated for 15 min. Cell pellet was then suspended again in lysis buffer and disrupted by a narrow-gauge syringe (1 ml) eight times. Cells were centrifuged for 5 min at 11,000 × g. Supernatant was removed (cytoplasmic fraction). Nuclei pellet was suspended in the extraction buffer (420 mM KCl) containing DTT and PI as mentioned above. After 30 min of rotation, tubes were centrifuged for 5 min at 21,000 × g. Supernatant was then conveyed to IP with FLAG antibody conjugated beads. Following overnight IP, protein sample buffer (lacking β-mercaptoethanol) was added and tubed were boiled at 95 °C for 5 min these samples were subjected to mass spectrometry analysis (Weizmann Institute of Science, Israel).

### Chromatin immunoprecipitation (ChIP)

Chromatin immunoprecipitation (ChIP) was performed as described^34^. Briefly, after formaldehyde cross-linking and six rounds of sonication (Bioruptor, Diagenode) 6 min each (30 s on/off), the samples were extracted with Chelex 100 resin (Bio-Rad) as described^34^ and dsDNA was measured by NanoDrop. Then, following this calculation, equal amount of protein-DNA complexes were pre-cleared overnight by transferring the sonicated samples onto 20 µl beads (nProtein A Sepharose 4 Fast Flow, GE) containing tubes. Immunoprecipitation with the indicated antibodies performed by overnight incubation. Reverse crosslinked (150 µl of elution buffer containing 1% SDS, 50 mM NaHCO_3_ and 140 mM NaCl were added to IP and input tubes, RNase was added to a final concentration of 0.2 μg/μl and incubated at 37 °C for an hour, shaking every 15 min. ProteinaseK was added to a final concentration of 0.2 μg/μl and incubated for another hour at 55 °C, shaking every 15 min. Supernatant was then transferred to new tubes and incubated for 4 h or overnight at 65 °C) purified DNA was amplified by real-time qPCR with specific primers for the BAX, NoxA, and PUMA promoters at their p53 binding site. The sequences of the primers used: BAX Fw 5′-TAATCCCAGCGCTTTGGAA-3′ Rev 5′-TGCAGAGACCTGGATCTAGCAA-3′, NoxA Fw 5′-CAGCGTTTGCAGATGGTCAA-3′ Rev 5‘-CCCCGAAATTACTTCCTTACAAAA-3′ and PUMA Fw 5′-CGTACATCGGTCGGTCTGTGTACG-3′ Rev 5′-CCAGACACCGGGACAGTCG-3′.

### RNA extraction and quantitative RT-PCR

Total RNA was extracted using NucleoSpin RNA (Macherey-Nagel) following manufacturer’s instructions. Extracted RNA (200 ng) was reverse-transcribed into cDNA using iScript cDNA Synthesis Kit (Bio-Rad), according to the manufacturer’s instructions. Real-time qPCR was carried out using the UPL probe library system (Roche). Samples were loaded in triplicates into 384-well plate and amplified in LightCycler 480 System (Roche). Expression levels were normalized with GAPDH following the 2-DDCt analyzation method^35^. The real-time qPCR primers were the following: BAX Fw 5′-CATCATGGGCTGGACATTG-3′ Rev 5′-GGGACATCAGTCGCTTCAGT-3′; NoxA Fw 5′-GGAGATGCCTGGGAAGAAG-3′ Rev 5′-CCTGAGTTGAGTAGCACACTCG-3′; PUMA Fw 5′- CGTACATCGGTCGGTCTGTGTACG-3′ Rev 5′- CCAGACACCGGGACAGTCG -3′; and GAPDH Fw 5′-CTACTAGCGGTTTTACGGGCG-3′ Rev 5′-TCGAACAGGAGGAGCAGAGAGCGA-3′.

### Apoptosis assay by flow cytometry (FACS) and immunofluorescence

Apoptosis detection assay was performed by staining the DOX treated HCT-116 cells with Annexin V-FITC and propidium iodide (PI) using a MEBCYTO Apoptosis kit (MBL, Nagoya). Briefly, cells were collected with trypsin, washed once with PBSx1 and then stained with 2 µl of Annexin-V FITC and 1 µl PI to each sample in 500 µl of binding buffer (100 mM HEPES, 140 mM NaCl, and 25 mM CaCl_2_, pH 7.4. After 15 min incubation in dark, samples were analyzed by flow cytometry (Guava® easyCyte flow cytometer). Apoptosis was defined as the total percentage of cells positive for both, PI and Annexin V. Similar protocol was used for the microscopic experiments. Briefly, cells were remained at their wells, washed once with PBSx1 followed by binding buffer addition with the mentioned volume of Annexin V and PI. Cells were incubated in dark, at room temperature for 20 min and then visualized under the microscope (EVOS FL cell imaging station Thermo Fisher).

### Statistical analysis

Statistical analysis was performed applying Student’s two-tailed *t*-test (unpaired) using GraphPad Prism software.

## Supplementary information

supplemental figure legends

Figure S1

Figure S2

Figure S3

Figures S4

Supplementary Table
