# Supplementary material for: SETD3 is a positive regulator of DNA-damage-induced apoptosis
Source: Cell Death Dis. 2019 Jan 25;10(2):74. doi: 10.1038/s41419-019-1328-4 (PMC6347638; doi:10.1038/s41419-019-1328-4)
Supplement: Supplementary file 1 — supplemental figure legends [file 41419_2019_1328_MOESM1_ESM.docx]

**SETD3 is a positive regulator of DNA-damage induced apoptosis**

Elina Abaev-Schneiderman^1,2^, Lee Admoni-Elisha ^1,2^ and Dan Levy^1,2#^

^1^The Shraga Segal Department of Microbiology, Immunology and Genetics and the ^2^National Institute for Biotechnology in the Negev, Ben-Gurion University of the Negev, P.O.B. 653, Be'er-Sheva 84105, Israel

^#^Correspondence should be addressed to D.L: [ledan@post.bgu.ac.il](mailto:ledan@post.bgu.ac.il)

**Supplementary figures**

**Fig S1: DMSO treatment staining.** FITC-Annexin V and PI staining of control and SETD3 KO cells. Cells were visualized under fluorescent microscope (scale bar signifies 100µM, pictures were taken under x20 magnification).

**Fig S2: DNA-damage induced apoptosis is SETD3 and methylation dependent.** FITC-Annexin V and PI staining of control, SETD3 KO#2 and KO#2 rescued (with WT or Y313A) cells after DOX treatment. Cells were visualized under fluorescent microscope (scale bar signifies 100µM, pictures were taken under x20 magnification).

**Fig S3: SETD3 positively regulates Etoposide and Abiplatin induced apoptosis.**

(A) FACS analysis of KO and control cells post Etoposide treatment or untreated. (B) Quantification of apoptotic cells percentage of 3 independent FACS analyses, *** p ≤0.001. (C) FACS analysis of KO and control cells post Abiplatin treatment or untreated. (D) Quantification of apoptotic cells percentage of 3 independent FACS analyses, ***p≤0.001.

**Fig S4: SETD3 catalytic activity is required for p53 recruitment to its target genes following DOX treatment.** (A) Chromatin immunoprecipitation (ChIP) assay of control and SETD3 KO HCT-116 cells treated with DOX. DNA fragments were immunoprecipitated with p53 antibody or beads. Values were compared to input samples. * p ≤0.05,**p ≤0.01, *** p ≤0.001. (B) Chromatin immunoprecipitation (ChIP) assay of control, SETD3 KO, SETD3 WT ~~rescue~~ and catalytic inactive (Y313A) SETD3 rescued cells treated with DOX. DNA fragments were immunoprecipitated with p53 antibody or beads. Values were compared to input samples. * p ≤0.05,** p ≤0.01, *** p ≤0.001.
