# Supplementary figures and images for: SETD3 is a positive regulator of DNA-damage-induced apoptosis
Source: Cell Death Dis. 2019 Jan 25;10(2):74. doi: 10.1038/s41419-019-1328-4 (PMC6347638; doi:10.1038/s41419-019-1328-4)

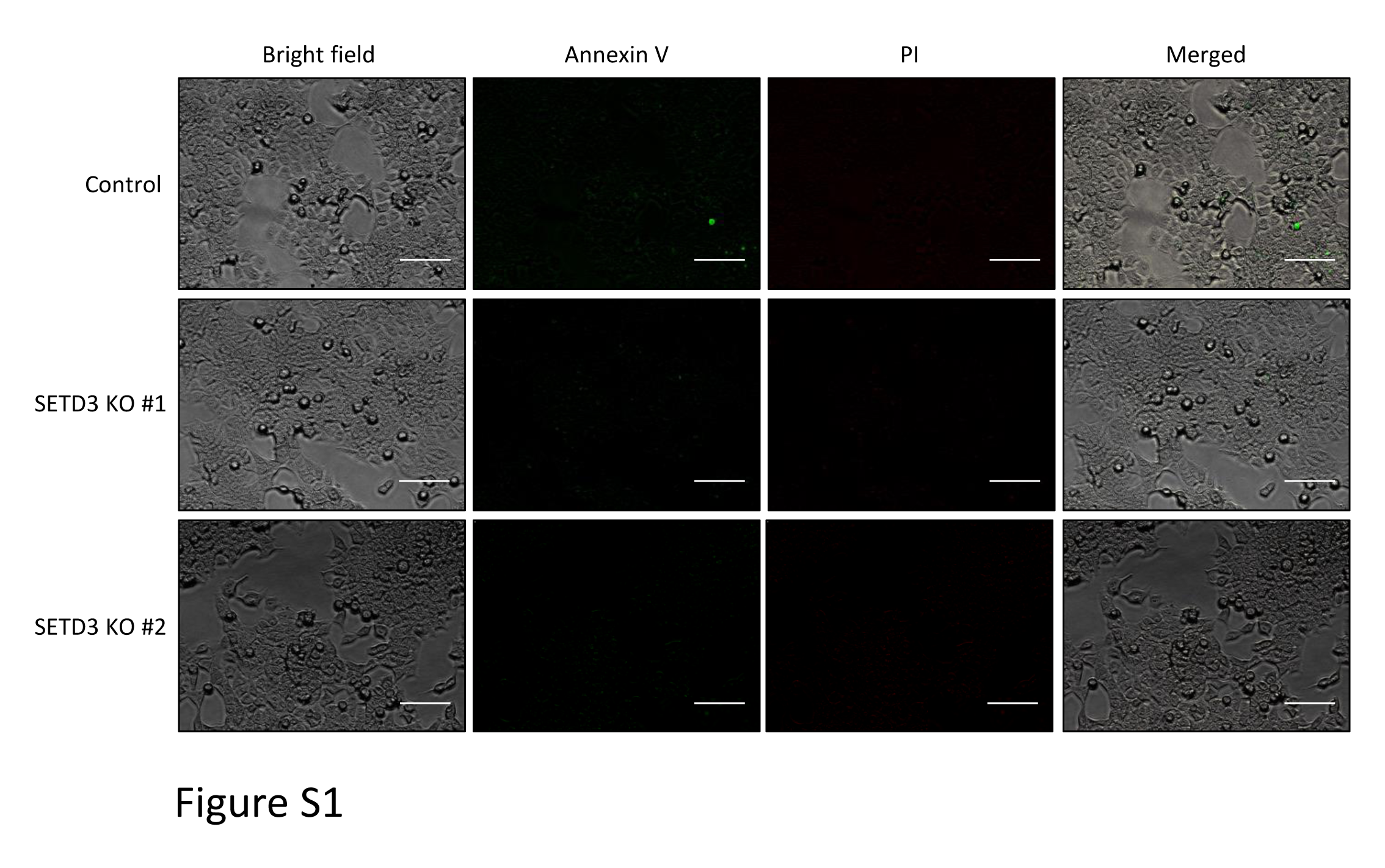

Supplement: Supplementary file 2 — Figure S1 [file 41419_2019_1328_MOESM2_ESM.tif]

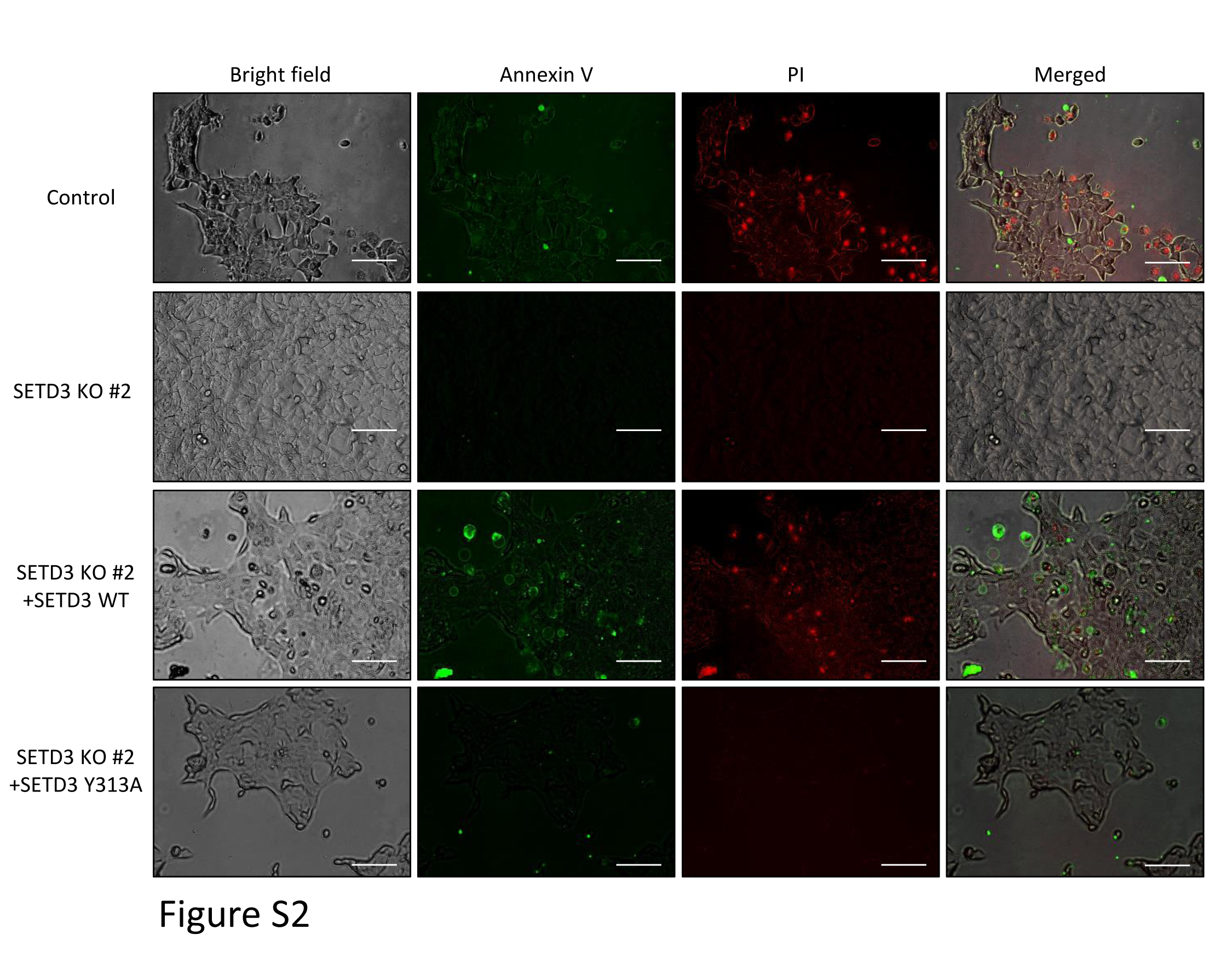

Supplement: Supplementary file 3 — Figure S2 [file 41419_2019_1328_MOESM3_ESM.tif]

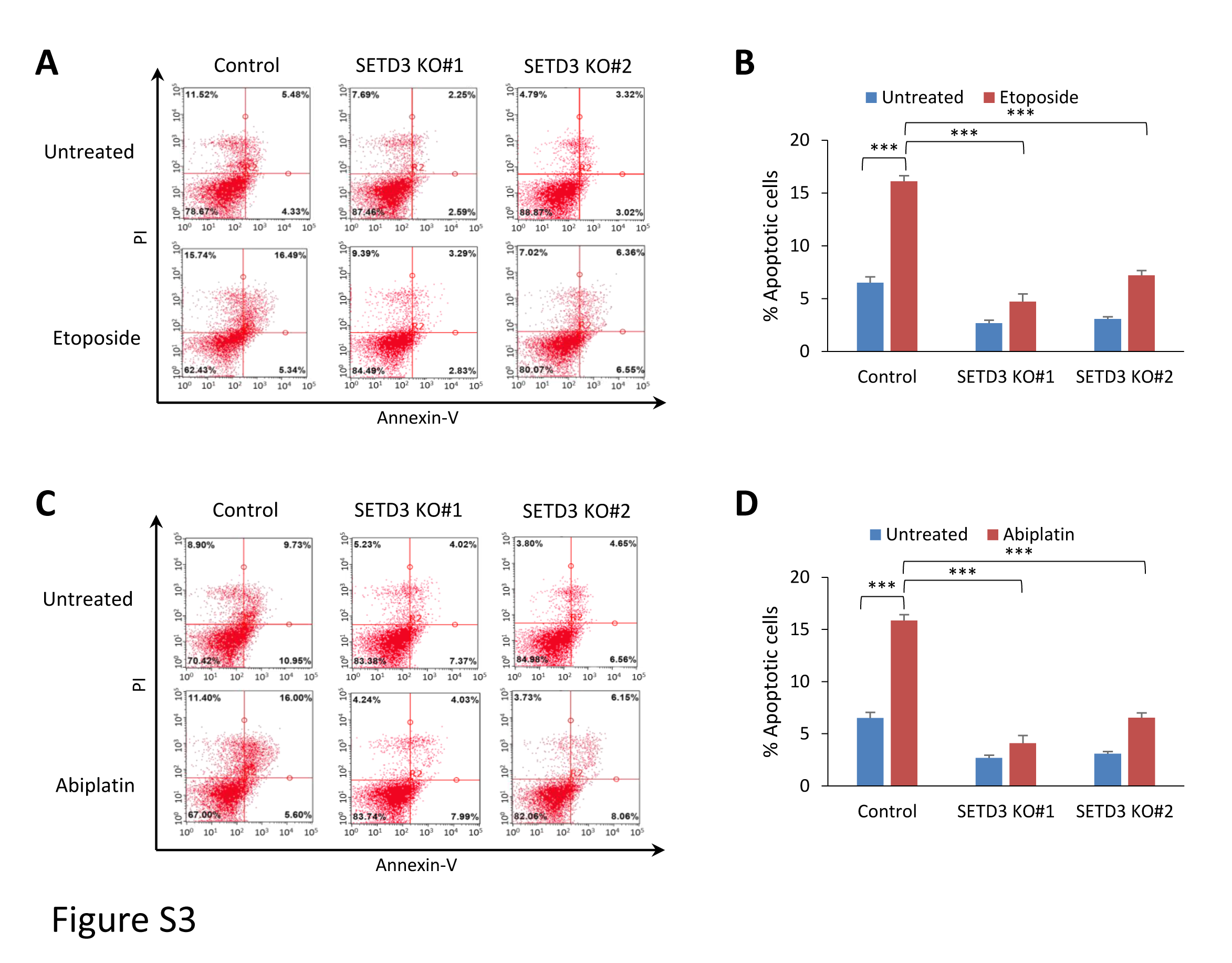

Supplement: Supplementary file 4 — Figure S3 [file 41419_2019_1328_MOESM4_ESM.tif]

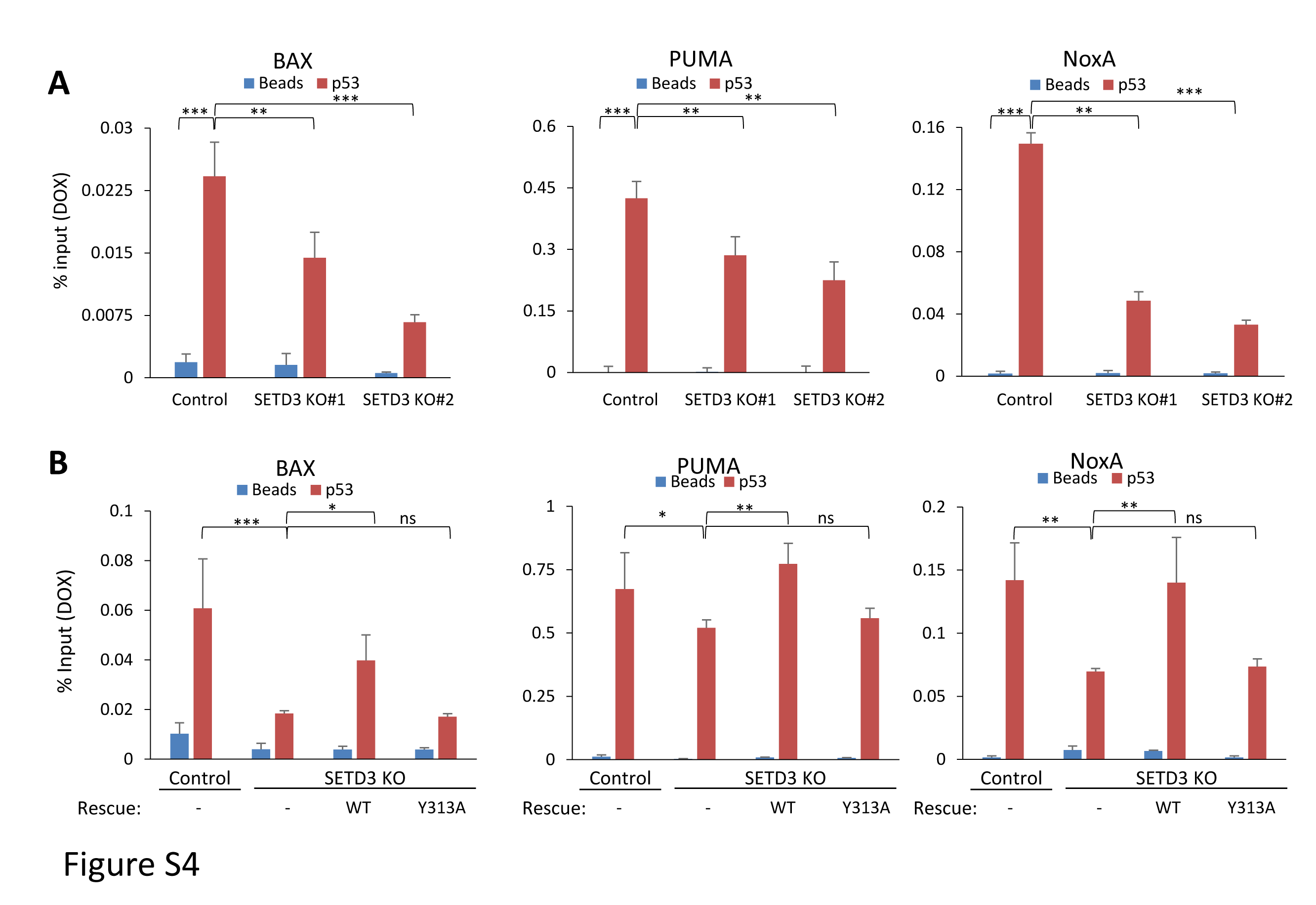

Supplement: Supplementary file 5 — Figures S4 [file 41419_2019_1328_MOESM5_ESM.tif]
