# Supplementary material for: SETD3 is a positive regulator of DNA-damage-induced apoptosis
Source: Cell Death Dis. 2019 Jan 25;10(2):74. doi: 10.1038/s41419-019-1328-4 (PMC6347638; doi:10.1038/s41419-019-1328-4)
Supplement: Supplementary file 6 — Supplementary Table [file 41419_2019_1328_MOESM6_ESM.pdf]

UNIPROT Accession	Gene Name	Description	Coverage	# Peptides	# PSMs	# Unique P	# AAs	MW [kDa]	calc. pI
U3KQT1	ESD	S-formylglutathione hydrolas	5.833333	1	1	1	120	13.101	8.69
Q9Y6V7	DDX49	Probable ATP-dependent RN/	2.070393	1	2	1	483	54.192	9.06
Q9Y6J9	TAF6L	TAF6-like RNA polymerase II	3.697749	1	2	1	622	67.772	8.97
Q9Y6B6	SAR1B	GTP-binding protein SAR1b C	16.16162	4	16	1	198	22.396	6.11
Q9Y446	PKP3	Plakophilin-3 OS=Homo sapie	2.007528	1	2	1	797	87.029	9.32
Q9Y3C7	MED31	Mediator of RNA polymerase	12.9771	1	2	1	131	15.795	8.54
Q9Y3B3	TMED7	Transmembrane emp24 dom	5.357143	1	2	1	224	25.156	6.89
Q9Y2H1	STK38L	Serine/threonine-protein kin	6.034483	2	5	1	464	53.968	6.81
Q9ULG1	INO80	DNA helicase INO80 OS=Hom	1.092545	1	2	1	1556	176.643	9.5
Q9ULC5	ACSL5	Long-chain-fatty-acid--CoA li	1.903367	1	2	1	683	75.942	6.92
Q9UKZ1	CNOT11	CCR4-NOT transcription com	2.352941	1	2	1	510	55.18	6.4
Q9UI14	RABAC1	Prenylated Rab acceptor prot	12.43243	1	1	1	185	20.635	7.34
Q9UHI6	DDX20	Probable ATP-dependent RN/	1.213592	1	2	1	824	92.183	6.95
Q9UHD2	TBK1	Serine/threonine-protein kin	1.508916	1	2	1	729	83.589	6.79
Q9UBD5	ORC3	Origin recognition complex s	2.531646	1	2	1	711	82.201	7.61
Q9P275	USP36	Ubiquitin carboxyl-terminal h	1.873327	1	2	1	1121	122.575	9.67
Q9P086	MED11	Mediator of RNA polymerase	16.23932	1	2	1	117	13.121	5.96
Q9NYB0	TERF2IP	Telomeric repeat-binding fac	7.26817	1	2	1	399	44.233	4.73
Q9NY12	GAR1	H/ACA ribonucleoprotein cor	3.225806	1	2	1	217	22.334	10.92
Q9NXV6	CDKN2AIP	CDKN2A-interacting protein	4.655172	2	4	2	580	61.088	9.01
Q9NXB0	MKS1	Meckel syndrome type 1 pro	1.788909	1	1	1	559	64.487	6.46
Q9NX58	LYAR	Cell growth-regulating nucle	3.693931	1	1	1	379	43.588	9.54
Q9NX24	NHP2	H/ACA ribonucleoprotein cor	6.535948	1	2	1	153	17.19	8.22
Q9NVN8	GNL3L	Guanine nucleotide-binding	2.233677	1	2	1	582	65.532	8.44
Q9NVJ2	ARL8B	ADP-ribosylation factor-like p	15.05376	3	8	1	186	21.525	8.43
Q9NVC6	MED17	Mediator of RNA polymerase	1.228879	1	2	1	651	72.845	7.44
Q9NUQ6	SPATS2L	SPATS2-like protein OS=Hom	3.584229	1	2	1	558	61.691	9.64
Q9NRY2	INIP	SOSS complex subunit C OS=	11.53846	1	1	1	104	11.418	9.25
Q9NRL2	BAZ1A	Bromodomain adjacent to zi	1.156812	1	2	1	1556	178.592	6.6
Q9NQ29	LUC7L	Putative RNA-binding proteir	9.703504	3	6	1	371	43.701	9.92
Q9HC07	TMEM165	Transmembrane protein 165	7.716049	1	2	1	324	34.883	7.02
Q9HBH0	RHOF	Rho-related GTP-binding prot	9.952607	2	4	2	211	23.61	8.65

Q9H4L7	SMARCAD1	SWI/SNF-related matrix-asso	1.169591	1	2	1	1026	117.328	5.55
Q9H1I8	ASCC2	Activating signal cointegrator	1.321004	1	2	1	757	86.306	5.16
Q9H1E3	NUCKS1	Nuclear ubiquitous casein an	14.81481	2	5	2	243	27.28	5.08
Q9H1B7	IRF2BPL	Interferon regulatory factor 2	6.281407	3	6	3	796	82.607	8.24
Q9H0D6	XRN2	5'-3' exoribonuclease 2 OS=H	3.052632	2	4	2	950	108.513	7.47
Q9H089	LSG1	Large subunit GTPase 1 homo	1.06383	1	1	1	658	75.178	6.38
Q9C0J8	WDR33	pre-mRNA 3' end processing	1.347305	1	1	1	1336	145.799	9.17
Q9BZJ0	CRNKL1	Crooked neck-like protein 1 C	1.415094	1	2	1	848	100.389	8
Q9BZ95	NSD3	Histone-lysine N-methyltrans	1.600557	1	1	1	1437	161.509	8.21
Q9BYJ9	YTHDF1	YTH domain-containing famil	9.660107	3	19	1	559	60.836	8.79
Q9BWE0	REPIN1	Replication initiator 1 OS=Ho	2.645503	1	2	1	567	63.534	9.98
Q9BSR8	YIPF4	Protein YIPF4 OS=Homo sapie	3.688525	1	2	1	244	27.065	4.65
Q9BRK5	SDF4	45 kDa calcium-binding prote	2.762431	1	2	1	362	41.78	4.86
Q9BQ75	CMSS1	Protein CMSS1 OS=Homo sap	4.659498	1	2	1	279	31.864	9.19
Q9BQ39	DDX50	ATP-dependent RNA helicase	1.221167	1	2	1	737	82.514	9.17
Q9BQ15	NABP2	SOSS complex subunit B1 OS=	10.42654	1	4	1	211	22.324	9.06
Q99986	VRK1	Serine/threonine-protein kin	6.818182	1	3	1	396	45.447	8.91
Q96ST2	IWS1	Protein IWS1 homolog OS=Ho	1.709402	1	2	1	819	91.899	4.69
Q96RE7	NACC1	Nucleus accumbens-associat	2.466793	1	2	1	527	57.222	5.74
Q96JM7	L3MBTL3	Lethal(3)malignant brain tum	1.666667	1	2	1	780	88.28	6.46
Q96II8	LRCH3	Leucine-rich repeat and calpo	1.544402	1	2	1	777	86.03	6.71
Q96HS1	PGAM5	Serine/threonine-protein pho	3.460208	1	2	1	289	31.985	8.68
Q96EP5	DAZAP1	DAZ-associated protein 1 OS=	3.685504	1	2	1	407	43.356	8.56
Q96DT7	ZBTB10	Zinc finger and BTB domain-c	0.918485	1	2	1	871	94.835	5.16
Q96A65	EXOC4	Exocyst complex component	3.38809	2	4	2	974	110.429	6.49
Q92736	RYR2	Ryanodine receptor 2 OS=Ho	0.181196	1	2	1	4967	564.206	6.07
Q92621	NUP205	Nuclear pore complex protei	0.745527	1	2	1	2012	227.776	6.19
Q92620	DHX38	Pre-mRNA-splicing factor ATP	1.385493	1	2	1	1227	140.415	6.54
Q92610	ZNF592	Zinc finger protein 592 OS=H	1.736385	1	2	1	1267	137.441	7.84
Q92597	NDRG1	Protein NDRG1 OS=Homo sap	3.553299	1	2	1	394	42.808	5.82
Q92576	PHF3	PHD finger protein 3 OS=Hom	0.784698	1	1	1	2039	229.339	6.96
Q92499	DDX1	ATP-dependent RNA helicase	2.162162	1	2	1	740	82.38	7.23
Q8WXD5	GEMIN6	Gem-associated protein 6 OS	7.784431	1	2	1	167	18.812	5.12
Q8WUA4	GTF3C2	General transcription factor 3	2.963776	1	2	1	911	100.616	7.31

Q8TEK3	DOT1L	Histone-lysine N-methyltrans	2.415181	2	3	2	1739	184.74	9.33
Q8TC12	RDH11	Retinol dehydrogenase 11 OS	4.402516	1	2	1	318	35.363	8.82
Q8TAT6	NPLOC4	Nuclear protein localization p	1.644737	1	2	1	608	68.077	6.38
Q8TA86	RP9	Retinitis pigmentosa 9 protei	9.049774	2	4	2	221	26.091	9.79
Q8NHQ9	DDX55	ATP-dependent RNA helicase	2.333333	1	2	1	600	68.503	9.25
Q8NFW8	CMAS	N-acylneuraminate cytidylylt	3.686636	1	2	1	434	48.349	7.93
Q8NE71	ABCF1	ATP-binding cassette sub-fan	4.733728	3	5	3	845	95.866	6.8
Q8NDT2	RBM15B	Putative RNA-binding protein	0.786517	1	2	1	890	97.147	9.85
Q8NCA5	FAM98A	Protein FAM98A OS=Homo sa	4.238921	1	2	1	519	55.366	9.03
Q8N9N7	LRRC57	Leucine-rich repeat-containin	2.92887	1	2	1	239	26.737	8.43
Q8N9M1	C19orf47	Uncharacterized protein C19	3.317536	1	2	1	422	44.718	10.11
Q8N183	NDUFAF2	Mimitin, mitochondrial OS=H	18.3432	2	3	2	169	19.844	8.97
Q8IYS2	KIAA2013	Uncharacterized protein KIAA	2.681388	1	1	1	634	69.113	8.19
Q8IX12	CCAR1	Cell division cycle and apopto	1.478261	1	2	1	1150	132.739	5.76
Q8IVF5	TIAM2	T-lymphoma invasion and me	0.823045	1	1	1	1701	189.985	7.21
Q86XP3	DDX42	ATP-dependent RNA helicase	1.066098	1	2	1	938	102.912	7.02
Q86UE4	MTDH	Protein LYRIC OS=Homo sapie	1.890034	1	2	1	582	63.799	9.32
Q7Z7H5	TMED4	Transmembrane emp24 dom	3.524229	1	2	1	227	25.926	8.28
Q7Z6E9	RBBP6	E3 ubiquitin-protein ligase RE	0.669643	1	2	1	1792	201.442	9.64
Q7RTS9	DYM	Dymeclin OS=Homo sapiens (	1.494768	1	2	1	669	75.887	5.86
Q7KZF4	SND1	Staphylococcal nuclease dom	1.208791	1	2	1	910	101.934	7.17
Q76L83	ASXL2	Putative Polycomb group pro	0.905923	1	2	1	1435	153.725	8.81
Q71F56	MED13L	Mediator of RNA polymerase	0.542986	1	2	1	2210	242.447	6.04
Q6ZU65	UBN2	Ubinuclein-2 OS=Homo sapie	1.039347	1	1	1	1347	145.998	9.19
Q6XZF7	DNMBP	Dynamin-binding protein OS=	0.380469	1	2	1	1577	177.236	5.39
Q6V1X1	DPP8	Dipeptidyl peptidase 8 OS=Ho	1.002227	1	2	1	898	103.293	5.8
Q6PD62	CTR9	RNA polymerase-associated p	1.449275	1	2	1	1173	133.42	6.77
Q68E01	INTS3	Integrator complex subunit 3	2.876318	1	2	1	1043	117.994	5.8
Q68CP9	ARID2	AT-rich interactive domain-co	1.253406	1	2	1	1835	197.268	7.42
Q5VWC4	PSMD4	26S proteasome non-ATPase	3.157895	1	2	1	380	41.053	4.81
Q5VTL8	PRPF38B	Pre-mRNA-splicing factor 38E	2.014652	1	2	1	546	64.429	10.54
Q5TGY3	AHDC1	AT-hook DNA-binding motif-c	4.054897	3	8	3	1603	168.245	9.04
Q5T8P6	RBM26	RNA-binding protein 26 OS=H	2.085402	2	4	2	1007	113.527	9.16
Q5SRQ6	CSNK2B	Casein kinase II subunit beta	3.846154	1	1	1	234	26.908	5.96

Q53F19	NCBP3	Nuclear cap-binding protein s	2.096774	1	2	1	620	70.549	5.73
Q4KMP7	TBC1D10B	TBC1 domain family member	2.59901	1	2	1	808	87.145	9.19
Q15773	MLF2	Myeloid leukemia factor 2 OS	3.629032	1	2	1	248	28.129	6.9
Q15554	TERF2	Telomeric repeat-binding fac	3.874539	1	2	1	542	59.557	9.35
Q15084	PDIA6	Protein disulfide-isomerase A	3.181818	1	2	1	440	48.091	5.08
Q14781	CBX2	Chromobox protein homolog	3.759398	1	2	1	532	56.046	10.01
Q14451	GRB7	Growth factor receptor-boun	6.203008	2	4	2	532	59.643	8.5
Q14157	UBAP2L	Ubiquitin-associated protein	1.379945	1	2	1	1087	114.465	7.11
Q13838	DDX39B	Spliceosome RNA helicase DD	8.64486	3	8	1	428	48.96	5.67
Q13823	GNL2	Nucleolar GTP-binding protei	2.46238	1	2	1	731	83.603	9.25
Q13751	LAMB3	Laminin subunit beta-3 OS=H	0.59727	1	2	1	1172	129.489	7.21
Q13601	KRR1	KRR1 small subunit processor	4.986877	2	4	2	381	43.638	9.77
Q13535	ATR	Serine/threonine-protein kin	0.491679	1	2	1	2644	301.172	7.43
Q13501	SQSTM1	Sequestosome-1 OS=Homo s	5.909091	1	2	1	440	47.657	5.22
Q13415	ORC1	Origin recognition complex s	1.74216	1	2	1	861	97.289	9.22
Q13347	EIF3I	Eukaryotic translation initiati	14.15385	2	5	2	325	36.479	5.64
Q13200	PSMD2	26S proteasome non-ATPase	1.321586	1	2	1	908	100.136	5.2
Q12962	TAF10	Transcription initiation factor	11.46789	1	2	1	218	21.698	6.57
Q12948	FOXC1	Forkhead box protein C1 OS=	2.169982	1	2	1	553	56.753	8.51
Q12888	TP53BP1	Tumor suppressor p53-bindir	1.064909	1	1	1	1972	213.443	4.7
Q12802	AKAP13	A-kinase anchor protein 13 O	0.497689	1	2	1	2813	307.359	5.24
Q12768	WASHC5	WASH complex subunit strum	1.121657	1	2	1	1159	134.201	6.98
Q10570	CPSF1	Cleavage and polyadenylation	2.772003	3	6	3	1443	160.782	6.4
Q04941	PLP2	Proteolipid protein 2 OS=Hor	8.552632	1	2	1	152	16.68	7.24
Q02952	AKAP12	A-kinase anchor protein 12 O	1.234568	1	2	1	1782	191.367	4.41
Q01804	OTUD4	OTU domain-containing prot	4.578097	3	6	3	1114	123.968	6.71
Q01664	TFAP4	Transcription factor AP-4 OS=	2.95858	1	2	1	338	38.702	5.87
P98161	PKD1	Polycystin-1 OS=Homo sapier	0.836626	1	2	1	4303	462.239	6.73
P78549	NTHL1	Endonuclease III-like protein	2.884615	1	2	1	312	34.368	9.67
P78347	GTF2I	General transcription factor I	4.308617	4	8	4	998	112.346	6.39
P68400	CSNK2A1	Casein kinase II subunit alpha	12.27621	3	5	3	391	45.115	7.74
P62937	PPIA	Peptidyl-prolyl cis-trans isom	4.242424	1	2	1	165	18.001	7.81
P62191	PSMC1	26S protease regulatory subu	5.454545	2	4	2	440	49.154	6.21
P61981	YWHAG	14-3-3 protein gamma OS=H	10.12146	3	8	2	247	28.285	4.89

P61020	RAB5B	Ras-related protein Rab-5B O	15.34884	3	10	1	215	23.692	8.13
P57721	PCBP3	Poly(rC)-binding protein 3 OS	17.52022	5	24	1	371	39.44	8.07
P55795	HNRNPH2	Heterogeneous nuclear ribor	18.04009	6	27	1	449	49.232	6.3
P53999	SUB1	Activated RNA polymerase II	11.81102	1	2	1	127	14.386	9.6
P53396	ACLY	ATP-citrate synthase OS=Hon	0.817439	1	2	1	1101	120.762	7.33
P52756	RBM5	RNA-binding protein 5 OS=Hc	1.840491	1	2	1	815	92.097	6.28
P52294	KPNA1	Importin subunit alpha-5 OS=	1.672862	1	1	1	538	60.184	5.01
P51659	HSD17B4	Peroxisomal multifunctional	3.668478	2	4	2	736	79.636	8.84
P51648	ALDH3A2	Fatty aldehyde dehydrogenas	2.474227	1	2	1	485	54.813	7.88
P51149	RAB7A	Ras-related protein Rab-7a O	5.31401	1	2	1	207	23.475	6.7
P50213	IDH3A	Isocitrate dehydrogenase [NA	6.284153	2	4	2	366	39.566	6.92
P49327	FASN	Fatty acid synthase OS=Homo	1.075269	2	3	2	2511	273.254	6.44
P48443	RXRG	Retinoic acid receptor RXR-ga	2.37581	1	2	1	463	50.838	7.62
P42285	MTREX	Superkiller viralicidic activity	2.495202	2	4	2	1042	117.729	6.52
P39748	FEN1	Flap endonuclease 1 OS=Hon	8.684211	3	5	3	380	42.566	8.62
P38646	HSPA9	Stress-70 protein, mitochond	1.767305	1	1	1	679	73.635	6.16
P35269	GTF2F1	General transcription factor I	3.868472	1	2	1	517	58.205	7.49
P32322	PYCR1	Pyrroline-5-carboxylate redu	3.448276	1	2	1	319	33.34	7.61
P30153	PPP2R1A	Serine/threonine-protein pho	1.697793	1	2	1	589	65.267	5.11
P30040	ERP29	Endoplasmic reticulum reside	2.681992	1	2	1	261	28.975	7.31
P28290	SSFA2	Sperm-specific antigen 2 OS=	1.350278	1	2	1	1259	138.3	5.19
P26006	ITGA3	Integrin alpha-3 OS=Homo sa	0.856327	1	2	1	1051	116.538	6.77
P20042	EIF2S2	Eukaryotic translation initiati	3.003003	1	2	1	333	38.364	5.8
P19784	CSNK2A2	Casein kinase II subunit alpha	2.571429	1	2	1	350	41.187	8.56
P17931	LGALS3	Galectin-3 OS=Homo sapiens	5.6	1	1	1	250	26.136	8.56
P17041	ZNF32	Zinc finger protein 32 OS=Ho	3.663004	1	2	1	273	31.009	9.35
P16401	HIST1H1B	Histone H1.5 OS=Homo sapie	25.22124	5	13	3	226	22.566	10.92
P13639	EEF2	Elongation factor 2 OS=Homo	3.729604	2	4	2	858	95.277	6.83
P12270	TPR	Nucleoprotein TPR OS=Homo	0.46551	1	2	1	2363	267.131	5.02
P12268	IMPDH2	Inosine-5'-monophosphate d	1.55642	1	2	1	514	55.77	6.9
P11940	PABPC1	Polyadenylate-binding protei	2.201258	1	2	1	636	70.626	9.5
P10588	NR2F6	Nuclear receptor subfamily 2	1.980198	1	2	1	404	42.952	7.78
P08651	NFIC	Nuclear factor 1 C-type OS=H	3.543307	1	2	1	508	55.64	8.38
P04350	TUBB4A	Tubulin beta-4A chain OS=Hc	41.21622	16	95	2	444	49.554	4.88

O96005	CLPTM1	Cleft lip and palate transmem	1.494768	1	1	1	669	76.048	6.3
O95983	MBD3	Methyl-CpG-binding domain	7.560137	3	7	1	291	32.823	5.34
O95816	BAG2	BAG family molecular chaper	3.791469	1	2	1	211	23.757	6.7
O95785	WIZ	Protein Wiz OS=Homo sapien	2.119927	2	4	2	1651	178.563	6.86
O95573	ACSL3	Long-chain-fatty-acid--CoA li	1.527778	1	2	1	720	80.368	8.38
O95251	KAT7	Histone acetyltransferase KA	4.582651	2	4	2	611	70.598	8.85
O95104	SCAF4	Splicing factor, arginine/serin	1.743679	2	4	1	1147	125.79	9.55
O75152	ZC3H11A	Zinc finger CCCH domain-con	3.45679	2	4	2	810	89.076	8.37
O75146	HIP1R	Huntingtin-interacting protei	1.310861	1	2	1	1068	119.315	6.67
O60664	PLIN3	Perilipin-3 OS=Homo sapiens	3.917051	1	2	1	434	47.046	5.44
O60506	SYNCRIP	Heterogeneous nuclear ribon	7.383628	3	8	2	623	69.56	8.59
O60216	RAD21	Double-strand-break repair p	1.584786	1	2	1	631	71.645	4.65
O15294	OGT	UDP-N-acetylglucosamine--p	1.529637	1	1	1	1046	116.85	6.7
O15226	NKRF	NF-kappa-B-repressing facto	1.594203	1	2	1	690	77.624	8.79
O14776	TCERG1	Transcription elongation regu	1.092896	1	2	1	1098	123.823	8.65
O14647	CHD2	Chromodomain-helicase-DNA	0.929978	2	6	1	1828	211.214	8.1
O00767	SCD	Acyl-CoA desaturase OS=Hon	6.963788	1	1	1	359	41.496	9
O00257	CBX4	E3 SUMO-protein ligase CBX4	1.428571	1	1	1	560	61.329	9.36
O00255	MEN1	Menin OS=Homo sapiens GN	2.601626	1	2	1	615	67.981	6.55
O00148	DDX39A	ATP-dependent RNA helicase	8.196721	3	8	1	427	49.098	5.68
J3QQJ0	SAP30BP	SAP30-binding protein (Fragr	4.615385	1	2	1	325	35.861	4.98
I3L504	EIF5A	Eukaryotic translation initiati	4.301075	1	2	1	186	20.49	5.25
I3L1Q2	BCL7C	B-cell CLL/lymphoma 7 prote	4.095563	1	1	1	293	32.816	6.46
H7C0N4	SF1	Splicing factor 1 (Fragment) (	9.444444	1	2	1	180	18.738	9.57
H7BZJ3	PDIA3	Protein disulfide-isomerase A	31.70732	3	7	1	123	13.511	7.3
H0YGF8	PLCD3	1-phosphatidylinositol 4,5-bis	27.92793	1	1	1	111	12.301	7.93
H0Y9X1	TMA16	Translation machinery-associ	6.198347	1	2	1	242	27.664	9.57
G5E9T8	GOSR1	Golgi SNAP receptor complex	7.843137	2	3	2	255	29.064	9.91
F8W0W8	PPP1CC	Serine/threonine-protein pho	5.722892	2	6	1	332	38.225	6.83
E7EX29	YWHAZ	14-3-3 protein zeta/delta (Fr	8.943089	2	6	1	246	28.019	4.92
E7ERK9	EIF2B4	Translation initiation factor e	2.573529	1	2	1	544	59.677	9.42
E7EMB3	CALM2	Calmodulin OS=Homo sapien	8.163265	1	2	1	196	21.675	4.56
E5KLJ5	OPA1	Dynamin-like 120 kDa protein	0.985222	1	2	1	1015	117.67	7.77
D6W592	HNRNPLL	Heterogeneous nuclear ribon	2.767528	1	2	1	542	60.061	7.85

D6RER5	SEPT11	Septin-11 OS=Homo sapiens	3.009259	1	1	1	432	49.777	6.68
D3DQV9	EIF4G2	Eukaryotic translation initiati	0.882029	1	1	1	907	102.265	7.14
C9JCC6	DRAP1	Dr1-associated corepressor O	4.716981	1	2	1	212	23.19	5.27
B5MCJ9	TRIM66	Tripartite motif-containing pr	2.730924	1	2	1	1245	137.57	6.7
B4E2Q0	ATP2C1	Calcium-transporting ATPase	1.259182	1	2	1	953	104.645	7.2
B4DLN1	N/A	cDNA FLJ60124, Foundly simi	2.714932	1	2	1	442	48.069	9.52
B3KTM8	MORF4L1	Mortality factor 4-like protein	2.873563	1	2	1	348	40.004	9.11
A0A0X1KG71	NELFB	Negative elongation factor B	3.025478	2	3	2	628	69.995	6.04
A0A0U1RQQ9	SCYL2	SCY1-like protein 2 OS=Homo	1.714898	1	2	1	933	104.046	8.22
A0A0C4DFX9	NELFA	Negative elongation factor A	8.719852	2	4	2	539	58.462	9.26
A0A087WUE4	LENG8	Leukocyte receptor cluster m	1.830664	1	2	1	874	95.485	9.32
A0A075B746	MRPS21	28S ribosomal protein S21, m	9.195402	1	2	1	87	10.681	9.92
A0A024QZP7	CDK1	Cell division cycle 2, G1 to S a	5.387205	1	2	1	297	34.06	8.4
